# Multiuser Chirp Spread Spectrum Transmission in an Underwater Acoustic Channel Applied to an AUV Fleet †

**DOI:** 10.3390/s20051527

**Published:** 2020-03-10

**Authors:** Christophe Bernard, Pierre-Jean Bouvet, Antony Pottier, Philippe Forjonel

**Affiliations:** SEACom Department, L@bisen Yncréa-Ouest, 29228 Brest CEDEX 2, France; christophe.bernard@isen-ouest.yncrea.fr (C.B.); antony.pottier@isen-ouest.yncrea.fr (A.P.); philippe.forjonel@isen-ouest.yncrea.fr (P.F.)

**Keywords:** underwater communications, multiple access, chirp spread spectrum, direct sequence spread spectrum, code-division multiple access (CDMA), time-division multiple access (TDMA)

## Abstract

The objective of this paper is to provide a multiuser transmission technique for underwater acoustic communication in the framework of an Autonomous Underwater Vehicle (AUV) fleet. By using a variant of a Hyperbolically Frequency-Modulated (HFM) signal, we describe a new family of transmission techniques called MultiUser Chirp Spread Spectrum (MU-CSS), which allows a very simple matched-filter-based decoding. These techniques are expected to provide good resilience against multiuser interference while keeping good robustness to Underwater Acoustic (UWA) channel impairments like Doppler shift. Their implementation for the UWA scenario is described, and the performance results over a simulated shallow-water UWA channel are analyzed and compared against conventional Code-Division Multiple Access (CDMA) and Time-Division Multiple Access (TDMA) transmission. Finally, the feasibility and robustness of the proposed methods are verified over the underWater AcousTic channEl Replay benchMARK (Watermark), fed by several channel responses from sounding experiments performed in a lake.

## 1. Introduction

The Underwater Acoustic (UWA) channel is one of the most challenging channels for data communications. Due to the low celerity of acoustic waves (c = 1500 m·$s^{-1}$), UWA channels are characterized by extensive multipath effects and large Doppler spreads. Moreover, frequency- dependent attenuation, temporal variations, and background noise limit the achievable data rate considerably [1,2]. On the other hand, Autonomous Underwater Vehicles (AUVs) are used for several marine applications, such as in the military field with anti-submarine warfare, in the science field with wreck exploration, or in the industrial field with offshore energy research. Nowadays, the concept of several AUVs working together within a fleet is an on-going research axis [3]. UWA communication with an AUV fleet is used to control vehicles (downlink) or to gather data from vehicles (uplink). The quality and reliability of communications are essential, mainly in shallow water areas for which the multipath effect is stronger, leading to extensive intersymbol interference.

Multiuser communication protocols in an UWA channel can be divided into two categories—random or deterministic protocols. In random protocols, the data rate cannot be predicted in advance due to the phenomenon of collisions between different users. A classical example of random protocol is ALOHA [4] and its variants [5], which use the long propagation delays to reduce the number of collisions and, consequently, to increase the data rate. Another example of random protocol is the Carrier Sense Multiple Access (CSMA) method [6], which is based on channel listening to avoid collisions. On the other side, deterministic protocols perform deterministic assignments of channel resources to the users so that their activity on the channel is predictable. The method we propose in this paper aims at building a new set of mutually orthogonal waveforms to be assigned to the users of an UWA channel so as to separate them easily at the receiver side. This consequently falls into the class of deterministic protocols. Traditional methods for deterministic multiuser UWA transmissions are inspired by radio communications and adapted to the UWA channel. As examples, we can cite the Time-Division Multiple Access (TDMA) [7], Frequency Divsion Multiple Access (FDMA) [8], Code-Division Multiple Access (CDMA) [9], and Multi-Carrier Code-Division Multiple Access (MC-CDMA) [10] transmissions. Typically, FDMA is considered inefficient, since the UWA channel has limited bandwidth and exhibits a large Doppler spread that requires guard frequency bands between users, leading to wasting of the data rate. MC-CDMA transmission schemes suffer from both time/frequency selectivity of the UWA channel and multiple-access interference, and require complex iterative equalizers. Consequently, in the following, we will focus only on the TDMA and CDMA strategies. TDMA allows several users to share the same frequency channel by dividing the signal into different time slots. Each user alternatively uses their own time slot to transmit data without interfering with other users. However, as the number of users increases, the waiting time per user increases and the user data rate decreases. In CDMA transmission, the different users transmit information data simultaneously through a different spreading sequence for each user. The disadvantage of this method lies in the multiuser interference provided by the non-orthogonality of spreading sequences, especially when the user communication channel is selective in time or in frequency. Moreover, this effect is increased when the interference power is much larger than the received signal power. This phenomenon is well known in mobile communication networks as the *near–far* problem. To cope with interference terms in CDMA, advanced equalization schemes can be invoked, such as multiuser detection [11] or the Multi-User Multiple-Input Multiple-Output (MU-MIMO) technique combined with Passive Phase Conjugation (PPC) [12], but at the price of a higher decoding complexity and a limited number of users. Recently, the authors of [13] proposed an alternative to CDMA and TDMA by using chirp waveforms for UWA multiuser communication. To reduce the multiuser interference, the Virtual Time Reversal Mirror (VTRM) technique is used with a Fractional Fourier Transform (FrFT) at the reception. However, this method requires an estimate of the different channels and is limited to four users because of interference.

In this paper, we describe a new transmission scheme based on Chirp Spread Spectrum (CSS), entitled MU-CSS, that we originally introduced in [14]. The basic idea consists of building a set of mutually orthogonal chirp-based waveforms that will be resistant to Doppler spread and Doppler shift. The objective is, on the one hand, to take benefit from the robustness of chirps against UWA channel impairments and, on the other hand, to use orthogonality to separate multiple users at the receiver side using a simple matched filter. With respect to [14], we derive three new methods for building MU-CSS that optimize mutual orthogonality between waveforms. By assuming an uplink scenario where a fleet of $N_{u}$ AUVs in motion needs to transmit data to a receiver situated at the sea surface, we provide a performance comparison of each method over simulated and experimental replay channels.

The paper is organized as follows: The system model and state of the art of multiuser transmissions are introduced in Section 2. The proposed MU-CSS multiuser schemes are presented in Section 3. Performance results of the proposed schemes against conventional multiuser transmissions are obtained in Section 4 by using a shallow-water UWA channel simulator derived from [15,16] and in Section 5 by using the Watermark replay channel [17] fed by experiments conducted in Ty-Colo lake, Saint-Renan, France. Finally, conclusions are drawn in Section 6.

In the following, ${\left||.|\right|}_{2}$ denotes the euclidean norm, 〈.〉 the scalar product, E{.} denotes the statistical expectation, ${\left(.\right)}^{*}$ the complex conjugate, and u*v denotes the convolution product between *u* and *v*.

## 2. Multiuser Transmission

### 2.1. System Model

#### Transmitted Signal

Let $d_{i,k}$ be the *k*-th symbol transmitted by the *i*-th user; we assume that $d_{i,k}$ belong to a unit-amplitude Phase Shift Keying (PSK) alphabet, and are differentially encoded such that:

(1) $$d_{i,k}=d_{i,k-1}\cdot b_{i,k}\;\mathrm{with}\;i\in \left[1,N_{u}\right],k\in \left[2,N_{s}\right],$$

where $b_{k}$ is the original PSK data symbol and $d_{i,0}$ is set to 1. Beforehand, the data symbols $b_{i,k}$ are protected by a Forward Error Correction (FEC) code, followed by a random interleaver. In the following, the FEC code type will be a half-rate convolutive code with code generator ${\left(133,171\right)}_{o}$. Moreover, $N_{u}$ denotes the number of users and $N_{s}$ the number of data symbols per frame. The choice of Differential Phase Shift Keying (DPSK) is motivated by the rapid fluctuation of the UWA channel and thus allows one to avoid the use of channel equalizers at the receiver side, which are sensitive to outdated channel estimations [18]. Thus, in an UWA communication channel with large delay spreads and rapid time variations, differential modulations are demonstrated to provide interesting performance and even outperform coherent modulation under certain conditions [19].

Let $g_{i}\left(t\right)$ be the transmit waveform associated to user *i* and $T_{s}$ the symbol duration; the baseband transmit signal for user *i* can be written as:

(2) $$s_{i}\left(t\right)=\sum_{k=1}^{N_{s}}d_{i,k}g_{i}\left(t-kT_{s}\right).$$

### 2.2. Underwater Multiuser Channel

By assuming that users are mobile with relative motion $v_{i}$, positive values of $v_{i}$ denote motion away from the receiver, while negative values denote motion toward the receiver; the received baseband signal is given by:

(3) $$r\left(t\right)=\sum_{i=1}^{N_{u}}\int_{-\infty }^{+\infty }h_{i}\left(\tau ,t\right)s_{i}\left(\left(1-a_{i}\right)\left(t-\tau \right)\right)e^{j2\pi f_{c}a_{i}\left(t-\tau \right)}d\tau +n\left(t\right),$$

where $f_{c}$ is the carrier frequency and $a_{i}=\frac{v_{i}}{c}$ is the Doppler scale factor. The UWA channel impulse response for the *i*-th user at time *t* is denoted by $h_{i}\left(\tau ,t\right)$, and n(t) represents the additive noise, assumed to be Gaussian and zero-mean.

#### User Decoding

When the Doppler shift can be estimated at the receiver, the Doppler effect is usually removed prior to decoding by resampling the received baseband signal and compensating phase rotation as follows [1]:

(4) $$z_{i}\left(t\right)=r\left(\frac{t}{1-a_{i}}\right)e^{-j2\pi f_{c}\left(\frac{a_{i}}{1-a_{i}}\right)t}.$$

By assuming perfect time synchronization, information data of the *i*-th user can be estimated by matched filtering $z_{i}\left(t\right)$ with the transmitted waveform of user *i*, followed by integration over a symbol duration [20]:

(5) $$\begin{array}{cc} {\hat{d}}_{i,k} & =\max_{k\frac{T_{s}}{2}\leq t\leq \left(k+1\right)\frac{T_{s}}{2}}\left[\int_{-\infty }^{+\infty }g_{i}^{*}\left(-u\right)z_{i}\left(t-u\right)du\right] \end{array}$$

(6) $$\begin{array}{cc} \; & =\int_{-\frac{T_{s}}{2}}^{\frac{T_{s}}{2}}g_{i}^{*}\left(t\right)z_{i}\left(t+kT_{s}\right)dt \end{array}$$

(7) $$\begin{array}{cc} \; & =\gamma_{i,k}d_{i,k}+\eta_{i,k}+w_{i,k}, \end{array}$$

where $\gamma_{i,k}$ denotes the bias of the decoder, $\eta_{i,k}$ the multiuser interference terms, and $w_{i,k}$ the additive noise terms; the exact expression of these three terms is provided in Appendix A.

### 2.3. Conventional Multiuser Transmission Schemes

#### 2.3.1. CDMA

The objective of CDMA is to break up a finite transmission spectrum so that multiple users can access it at the same time. To accomplish time multiplexing, a code, chosen in a set of mutually orthogonal spreading codes, is assigned to each user [21]. For the *i*-th user, the transmitted waveform is expressed by:

(8) $$g_{i}\left(t\right)=c_{i}\left(t\right)=\sum_{l=0}^{N_{SF}-1}c_{i,l}\phi \left(t-lT_{c}\right),$$

where $\left[c_{i,1},c_{i,2},\ldots ,c_{i,N_{SF}}\right]$ is the spreading code of length $N_{SF}$, $T_{c}$ is the chip duration, $N_{SF}$ is the spreading factor, and ϕ(t) is the pulse-shaping filter chosen as a Square Root Raised Cosine (SRRC) filter [20]. Since we are in an uplink scenario, the CDMA system is asynchronous, and spreading codes are chosen as Pseudo-Noise (PN) sequences generated pseudo-randomly such that their autocorrelation functions tend to Dirac functions as $N_{SF}$ grows, the mutual cross-correlation tends to zero.

At the receiver side, if $T_{s}>\tau_{max}$, where $\tau_{max}$ denotes the Root Mean Square (RMS) channel delay spread, and if the communication channel is constant over a symbol duration $T_{s}$, the autocorrelation properties and quasi-orthogonality between users of PN codes lead the term $\eta_{i,k}$ in (7) to become negligible compared to $\gamma_{i,k}$, and thus allow each user to be decoded separately [20].

#### 2.3.2. TDMA

**Figure 1:**
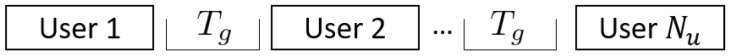
Scheme of Time-Division Multiple Access (TDMA).

In a TDMA approach, the users are time-multiplexed, as depicted in Figure 1. The time slot assigned to one user is made of a frame slot of $N_{s}T_{s}$ seconds, followed by a guard interval of duration $T_{g}$ so as to absorb multiuser interference. In order to deal with the frequency selectivity of the UWA channel, Direct Sequence Spread Spectrum (DSSS) signaling, with the same modulation parameters as CDMA, is chosen for each user such that the TDMA and CDMA approaches are equivalent in the single user scenario. The baseband received signal and the decoding process are given by particularizing (2) and (8) respectively with $N_{u}=1$. One can note that a more spectrally efficient transmission scheme could be chosen for TDMA (see [22], for example) but at the price of higher complexity at the receiver side. Moreover, a higher spectral efficiency signaling scheme would make the comparison with CDMA difficult, especially in the single-user case.

## 3. MU-CSS Scheme

### 3.1. Generalities

**Figure 2:**
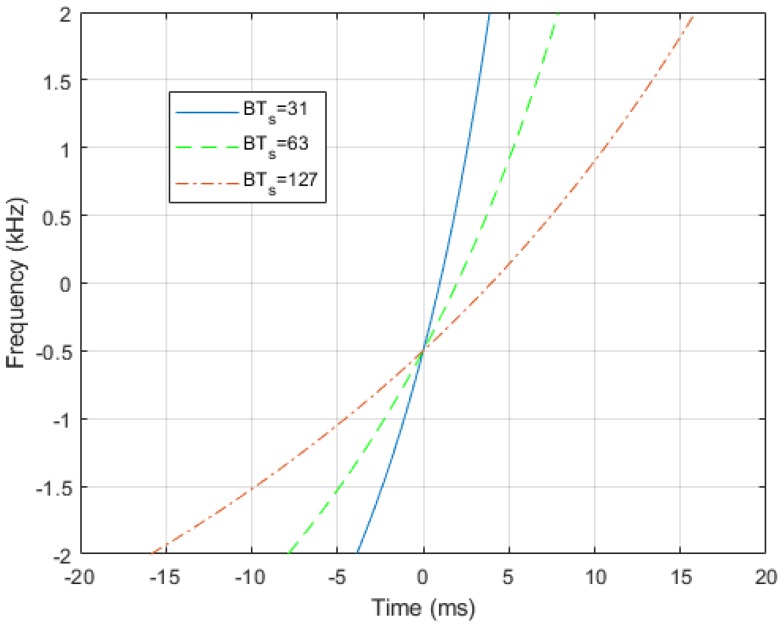
Instantaneous frequency of the Hyperbolically Frequency-Modulated (HFM) waveform with $BT_{s}=31$, 63, 127.

Through the use of frequency-swept signals, which are resilient to the detrimental effects of the UWA channel, the CSS modulation technique offers robust performance with a very simple matched filtering-based decoder that makes such a communication scheme particularly well adapted to the UWA communication channel [23,24]. In the CSS system, a broad spectrum is occupied to modulate the information in order to achieve high processing gain and multipath resolution to the detriment of the spectral efficiency. In the following, we construct three multiuser schemes based on CSS signaling and, more precisely, on a Hyperbolically Frequency-Modulated (HFM) signal given by:

(9) $$x\left(t\right)=\left\{\begin{array}{cc} \cos(-2\pi \left(k\log\left(1-\frac{t}{t_{0}}\right)+\frac{f_{l}+f_{h}}{2}\right)) & \mathrm{if}\;\frac{-T_{s}}{2}\leq t\leq \frac{T_{s}}{2}\\ 0 & \mathrm{otherwise}, \end{array}\right.$$

where $t_{0}=\frac{T_{s}\left(f_{h}+f_{l}\right)}{2(f_{h}-f_{l})}$, $k=\frac{T_{s}f_{l}f_{h}}{f_{h}-f_{l}}$, $f_{l}\leq f_{h}$, and $T_{s}$ is the duration of the HFM signal, whose instantaneous frequency is provided in Figure 2, with $f_{h}=B/2$ and $f_{l}=-B/2$, where B=4 kHz and $T_{s}=7.75$, 15.75, and 31.75 ms.

The basic idea of MU-CSS consists of building an orthogonal basis of signals $e_{i}\left(t\right)$ thanks to the Gram–Schmidt process, where the waveform $e_{i}\left(t\right)$ is assigned to *i*-th user with $i\in [1,N_{u}]$. The initial orthogonality between waveforms is brought by the combination of the HFM signals with orthogonal spreading sequences that are chosen as a Walsh–Hadamard code [21]. The set of spreading codes allows users to be differentiated at the receiver side, while the HFM waveform provides robustness against Doppler and delay spreads.

### 3.2. MU-CSS Gram–Schmidt Iterated

In this method, an iterative process is used to improve the mutual orthogonality between the chirp waveforms as well as the immunity against channel impairments.

Let $e_{i}^{\left(l\right)}\left(t\right)$ denote the waveform corresponding to the *i*-th user with $i\in \{1,2,\ldots ,N_{u}\}$ at iteration $l\in \{1,\ldots ,N_{IT}\}$. The process is based on the Gram–Schmidt method [25], as follows, for i>0:

(10) $$e_{i}^{\left(l\right)}\left(t\right)=c_{i}\left(t\right)+\alpha_{i}^{\left(l\right)}e_{i-1}^{\left(l\right)}\left(t\right),$$

where:

(11) $$\alpha_{i}^{\left(l\right)}=-\frac{\langle c_{i}\left(t\right),e_{i-1}^{\left(l\right)}\left(t\right)\rangle }{\left|\right|e_{i-1}^{\left(l\right)}\left(t\right){\left|\right|}_{2}^{2}}=-\frac{\int_{\frac{-T_{s}}{2}}^{\frac{T_{s}}{2}}c_{i}\left(t\right)e_{i-1}^{(l)*}\left(t\right)dt}{\left|\right|e_{i-1}^{\left(l\right)}\left(t\right){\left|\right|}_{2}^{2}},$$

with $c_{i}\left(t\right)$ given by Equation (8). At the first iteration, we set $e_{0}^{\left(1\right)}\left(t\right)=x\left(t\right)$, where x(t) is defined in (9) and for l>1, i>0:

(12) $$c_{i}\left(t\right)=e_{i}^{\left(l-1\right)}\left(t\right).$$

The final waveform assigned to each user is obtained after $N_{IT}$ iterations of the abovementioned process by setting $g_{i}\left(t\right)=e_{i}^{\left(N_{IT}\right)}\left(t\right)$. The orthogonality between the different $e_{i}^{\left(l\right)}\left(t\right)$ and the choice for the value of $\alpha_{i}^{\left(l\right)}$ are justified in Appendix B using the Gram–Schmidt procedure.

### 3.3. MU-CSS Gram–Schmidt Multiplication

In this method, the combination with the HFM is made by multiplying it with the spreading sequence while applying the Gram–Schmidt iteration process to ensure orthogonality. We start from:

(13) $$e_{i}\left(t\right)=c_{i}\left(t\right)+\alpha_{i}e_{i-1}\left(t\right)\;\mathrm{with}\;i\in \left[1,N_{u}\right]$$

with $\alpha_{i}$ defined in (11). Then, we build:

(14) $${\tilde{e}}_{i}\left(t\right)={\bar{e}}_{i}\left(t\right)+\beta_{i}{\tilde{e}}_{i-1}\left(t\right),$$

where ${\bar{e}}_{0}\left(t\right)={\tilde{e}}_{0}\left(t\right)=x\left(t\right)$ (this signal will be excluded from the set later) and for i>0:

(15) $${\bar{e}}_{i}\left(t\right)=x\left(t\right)e_{i}\left(t\right).$$

Moreover:

(16) $$\beta_{i}=-\frac{\langle {\bar{e}}_{i}\left(t\right),{\tilde{e}}_{i-1}\left(t\right)\rangle }{\left|\right|{\tilde{e}}_{i-1}\left(t\right){\left|\right|}_{2}^{2}}=-\frac{\int_{\frac{-T_{s}}{2}}^{\frac{T_{s}}{2}}{\bar{e}}_{i}\left(t\right){\tilde{e}}_{i-1}^{*}\left(t\right)dt}{\left|\right|{\tilde{e}}_{i-1}\left(t\right){\left|\right|}_{2}^{2}}.$$

The final waveform assigned to each user is obtained by setting $g_{i}\left(t\right)={\tilde{e}}_{i}\left(t\right)$.

### 3.4. MU-CSS Gram–Schmidt Insertion

In this last variant, we combine the previous method with the insertion of a HFM signal at regular intervals, such as:

(17) $${\bar{e}}_{i}\left(t\right)=\left\{\begin{array}{cc} x(t) & \mathrm{if}\;i=kp\;\mathrm{with}\;k\in N^{*}\\ x\left(t\right)e_{i}\left(t\right) & \mathrm{else}, \end{array}\right.$$

where *p* is the insertion step. The idea is to try to improve the robustness of the different waveforms. To impose orthogonality between spread signals, we simply apply Equations (13) and (14), and finally get $g_{i}\left(t\right)={\tilde{e}}_{i}\left(t\right)$.

## 4. Simulation Results

### 4.1. Underwater Acoustic Channel Simulator

For the simulation comparisons, we consider the UWA channel simulator provided by [15] based on a stochastic model. The time-varying transfer function for the *i*-th user is given by:

(18) $$H_{i}\left(f,t\right)={\bar{H}}_{i}\left(f\right)\sum_{p}h_{i,p}\gamma_{i,p}\left(f,t\right)e^{-j2\pi f\tau_{i,p}\left(t\right)},$$

where ${\bar{H}}_{i}\left(f\right)$ is the transfer function of direct path, $h_{i,p}$ is the relative path gain, $\gamma_{i,p}\left(f,t\right)$ represents the scattering coefficient modeled by a complex-valued Gaussian processes, whose statistics reflect the time coherence of the channel, and $\tau_{i,p}\left(t\right)$ denotes time-varying delay of the *p*-th path and can be expressed as:

(19) $$\tau_{i,p}\left(t\right)={\bar{\tau }}_{i,p}-\left({\bar{a}}_{i}+a_{i,p}\right)t,$$

where ${\bar{\tau }}_{i,p}$ is the average delay of path *p*, and ${\bar{a}}_{i}$ represents the mean Doppler shift induced by the motion of the *i*-th AUV relative to the receiver. In the following, we will assume that ${\bar{a}}_{i}$ is known at the receiver side and compensated. Moreover, $a_{i,p}$ is the residual Doppler factor that captures resulting motion-induced time scaling on the *p*-th path. Coefficients $a_{i,p}$ are assumed to be constant over a frame and to follow a zero-mean Gaussian distribution with variance $\sigma_{a}^{2}$. Time variations of $\gamma_{i,p}\left(f,t\right)$ and $\tau_{i,p}\left(t\right)$ lead to Doppler spread effects [15].

### 4.2. System Parameters

**Figure 3:**
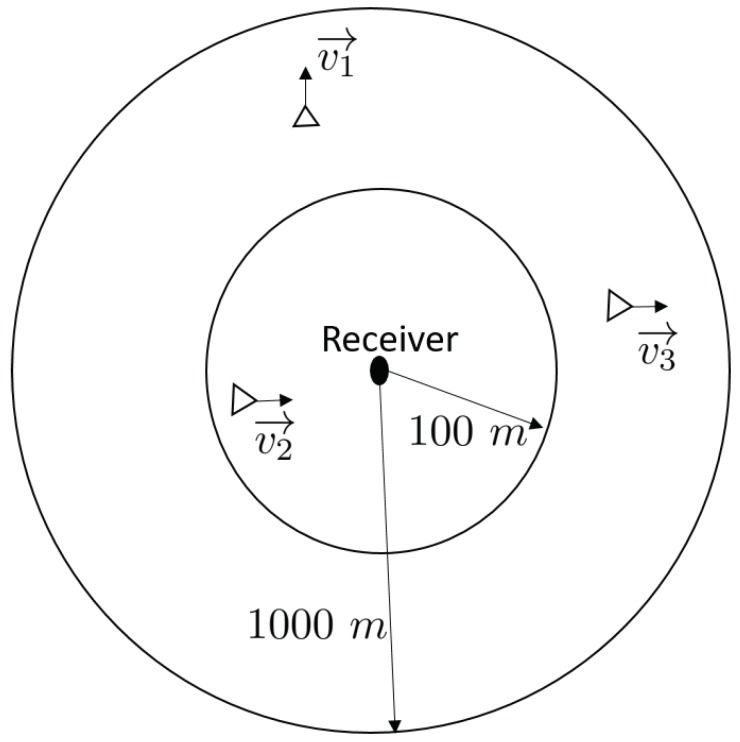
Scheme of the simulated system.

**Figure 4:**
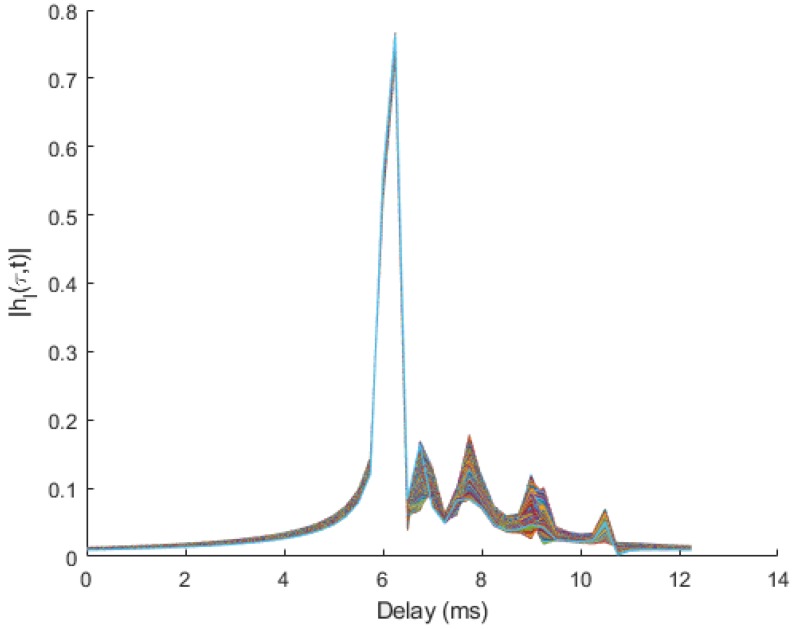
Evolution of the simulated time-varying channel impulse response over one frame based on the parameters provided in Table 1.

The chosen model represents a short-range UWA transmission with a 10 m water depth at a center frequency of 23 kHz over a 4 kHz bandwidth. Each AUV is assumed to be at the same depth of 1 m. At the beginning of the simulations, the range between each AUV and the receiver is randomly selected in the interval [0.1,1] km, modeling a fleet situating in a circular area (Figure 3). The channel model parameters are summarized in Table 1, whereas the transmission system parameters are provided in Table 2. The symbol duration is set according to the channel delay spread such that $T_{s}>\tau_{max}$ and is fixed identically for all protocols. The evolution of the simulated channel impulse response $|h_{i}\left(\tau ,t\right)|$ over one frame is provided in Figure 4.

### 4.3. Orthogonality Verification

To verify the orthogonality of the proposed waveform, we compute the Signal-to-Interference-plus-Noise Ratio (SINR) obtained after matched filtering. Following (7), for user *i*, we have:

(20) $$\mathrm{SINR}=\frac{E\left\{|\gamma_{i,k}{|}^{2}\right\}}{E\left\{|\eta_{i,k}{|}^{2}\right\}+E\left\{|w_{k}{|}^{2}\right\}}.$$

Simplifying (A2), (A3), and (A4) in the case of static AUV motion (i.e., $a_{i}=0$) and very small channel delay spread compared to the symbol duration (i.e., $T_{s}>>\tau_{max}$), the last equation becomes:

(21) $$\mathrm{SINR}=\frac{|\int_{-\frac{T_{s}}{2}}^{\frac{T_{s}}{2}}g_{i}^{*}\left(t\right)\left(\int_{-\infty }^{+\infty }h_{i}\left(t,\tau \right)g_{i}\left(t-\tau \right)d\tau \right)dt{|}^{2}}{|\sum_{\begin{array}{c} j=1\\ j\neq i \end{array}}^{N_{u}}\int_{-\frac{T_{s}}{2}}^{\frac{T_{s}}{2}}g_{i}^{*}\left(t\right)\left(\int_{-\infty }^{+\infty }h_{j}\left(t,\tau \right)g_{j}\left(t-\tau \right)d\tau \right)dt{|}^{2}+E\left\{|\int_{-\frac{T_{s}}{2}}^{\frac{T_{s}}{2}}g_{i}^{*}\left(t\right)n\left(t\right)dt{|}^{2}\right\}}.$$

**Figure 5:**
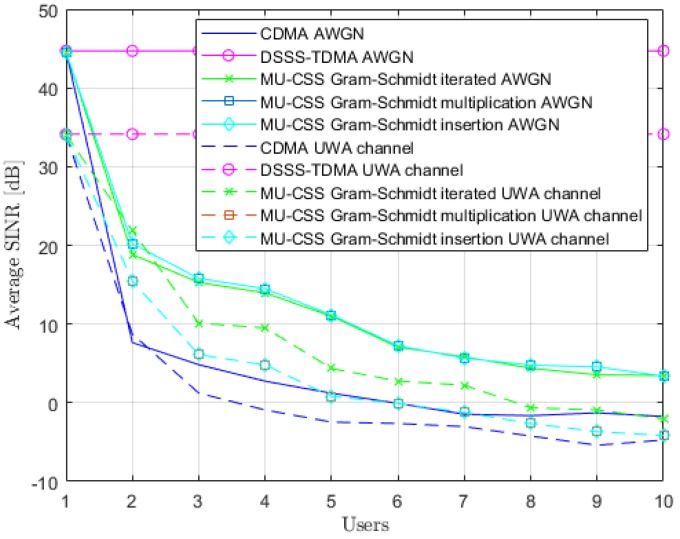
Average Signal-to-Interference-plus-Noise Ratio (SINR) for different waveforms over the Additive White Gaussian Noise (AWGN) and time-varying UWA channels with static users, SNR=30 dB.

In Figure 5, we numerically compute the SINR by using (21) and the system parameters depicted in Table 2 over an Additive White Gaussian Noise (AWGN) channel and over the time-varying UWA channel with static users described in Section 4.1. Comparisons are performed between MU-CSS, CDMA, and TDMA transmissions. At the $N_{u}=1$ user, since there are no interference terms, all of the transmission techniques have the same SINR after matched filter decoding, which is equal to channel Signal-to-Noise Ratio (SNR) added to the spreading gain in the case of AWGN channel. Naturally, as the the number of users increases, SINR decreases due to the growing importance of the interference terms, except for the TDMA case, for which the interference terms are absent whatever the number of users, thanks to time multiplexing. In both the AWGN and UWA channels, MU-CSS transmissions outperform CDMA, demonstrating that the Gram–Schmidt-based construction method provides good orthogonality properties for MU-CSS waveforms. This SINR gap is mainly explained by the use of PN sequences in CDMA that are not perfectly orthogonal (but only quasi-orthogonal), while MU-CSS employs waveforms that are orthogonal, owing to the Gram–Schmidt process. Obviously, this SINR gap could be erased in AWGN by the use of orthogonal codes like Walsh–Hadamard sequences for CDMA; however, such codes are not suitable in the uplink scenario.

### 4.4. Performance Metrics

As performance metrics, we consider the average effective data rate per user, defined for each transmission technique as follows:

(22) $$D_{e}^{\mathrm{CDMA}}=\frac{R_{C}\log_{2}M}{N_{SF}.T_{c}}\cdot \left(1-\mathrm{FER}\right)\;\left[\mathrm{bps}\right]$$

(23) $$D_{e}^{\mathrm{TDMA}}=\frac{R_{C}\log_{2}M}{N_{u}N_{SF}T_{c}+\left(N_{u}-1\right)T_{g}}\cdot \left(1-\mathrm{FER}\right)\;\left[\mathrm{bps}\right]$$

(24) $$D_{e}^{\mathrm{MU}-\mathrm{CSS}}=\frac{R_{C}\log_{2}M}{T_{h}}\cdot \left(1-\mathrm{FER}\right)\;\left[\mathrm{bps}\right],$$

where *M* is the size of the DPSK constellation, $R_{C}$ is channel coding rate, and FER is the Frame Error Rate. A frame is considered erroneous when at least one bit per frame after channel decoding is erroneous.

### 4.5. Static Channel

**Figure 6:**
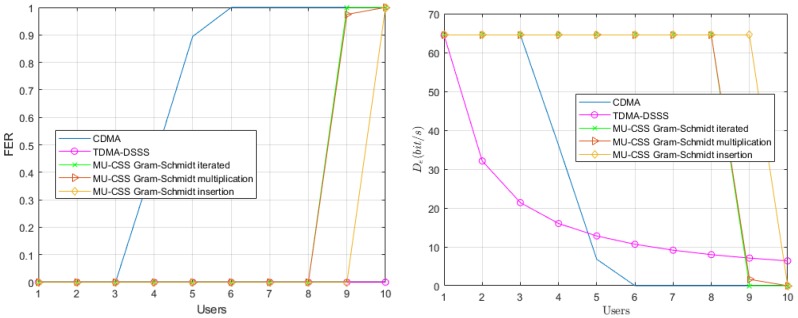
Average Frame Error Rate (FER) performance (**left**) and effective data rate (**right**) versus the number of users over the static simulated UWA channel model.

In a first step, we consider a static UWA channel leading to only frequency-selective fading. This yields the constant parameters $\gamma_{p}\left(f,t\right)$ and $\tau_{p}\left(t\right)$ in time in Equation (18). The Frame Error Rate (FER) performance and effective data rate of each transmission technique over the modeled shallow-water acoustic channel are provided in Figure 6.

In the single-user scenario, the three transmission techniques have an FER of 0, and, as expected, the FER of TDMA remains unchanged when the number of users increases. With more than four users, the interfering terms of the CDMA, expressed in Equation (7) by the quantity $\eta_{i,k}$, make the decoding of each user impossible. On the other side, the largest number of users that can be handled by the MU-CSS is eight or nine, depending on the method. The fact that MU-CSS outperforms CDMA is mainly explained by the better orthogonality properties of the MU-CSS waveforms.

### 4.6. Time-Varying Channel and Static Users

**Figure 7:**
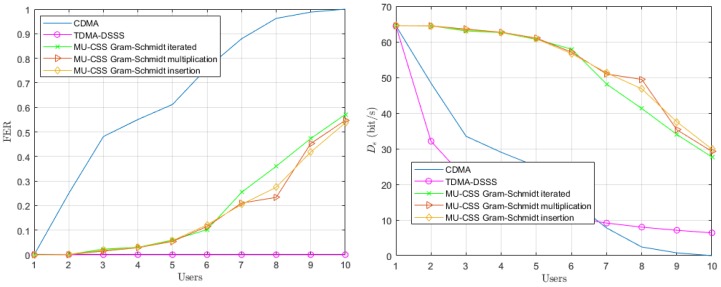
Average FER performance (**left**) and effective data rate (**right**) versus number of users over the time-varying simulated UWA channel model with static users.

In a second step, we consider a time-varying channel model, where Doppler spread effect is provided in the Equation (18) by the $\gamma_{i}\left(f,t\right)$ and $\tau_{i,p}\left(t\right)$ coefficients. In this scenario, we assume that all users are static, yielding ${\bar{a}}_{i}=0$ in Relation (19). The performance of the time-varying channel with static users is depicted in Figure 7.

The Doppler spread effect provided by multipath time variations leads to an FER increase of both the CDMA and MU-CSS transmissions, while the decoding performance TDMA still remains error-free. In fact, the TDMA transmission is not affected by multiuser interference, but only by UWA channel time and frequency selectivity, while CDMA and MU-CSS suffer from multiuser interference in addition to the UWA channel selectivity. The MU-CSS transmissions have the best effective data rate compared to CDMA because the HFM signal makes the spreading signals resistant against channel impairments, such as Doppler spread. Among the MU-CSS transmission technique, the Gram–Schmidt iterated method appears to be slightly less robust than the other methods.

### 4.7. Time-Varying Channel and Mobile Users

In a last step, we consider a time-varying channel model with mobile AUVs whose speeds are randomly selected in the interval [−2,2] m/s at each frame and for each user. The motion-induced Doppler shift is assumed to be perfectly known and compensated at the reception for each user *i*. According to (4), since each user has different speed, Doppler compensation of user *i* will increase the power of the interference terms. However, in practice, Doppler shift is unknown and must be estimated prior to decoding [26].

**Figure 8:**
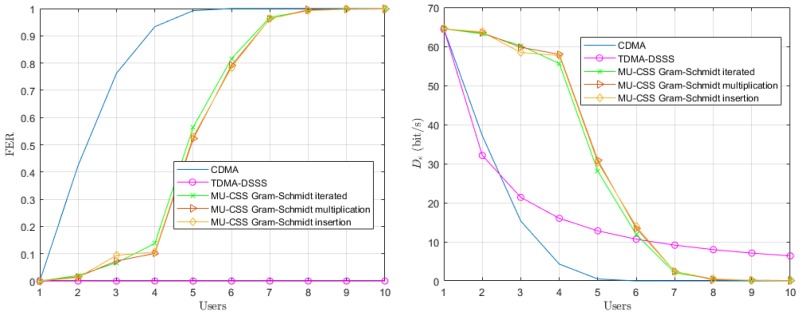
Average FER performance (**left**) and effective date rate (**right**) versus number of users over the time-varying UWA channel model with mobile users.

The performance of the time-varying UWA channel with mobile users is shown in Figure 8. In the single-user scenario, the three transmission techniques provide a FER of 0% and, as expected, the FER of TDMA remains unchanged when the number of users increases. Both the CDMA and MU-CSS transmissions are severely impacted by motion-induced Doppler shift, since Doppler shift correction for an user also applies to other users, according to Equation (4). However, the MU-CSS transmission still outperforms CDMA, which might be explained by the MU-CSS construction, which provides both an orthogonality enhancement and a better robustness against Doppler shift. Beyond six users, the TDMA approach is more efficient in terms of data rate.

## 5. Experimental Results

### 5.1. Channel Sounding

#### Ty-Colo Lake of Saint-Renan (France)

**Figure 9:**
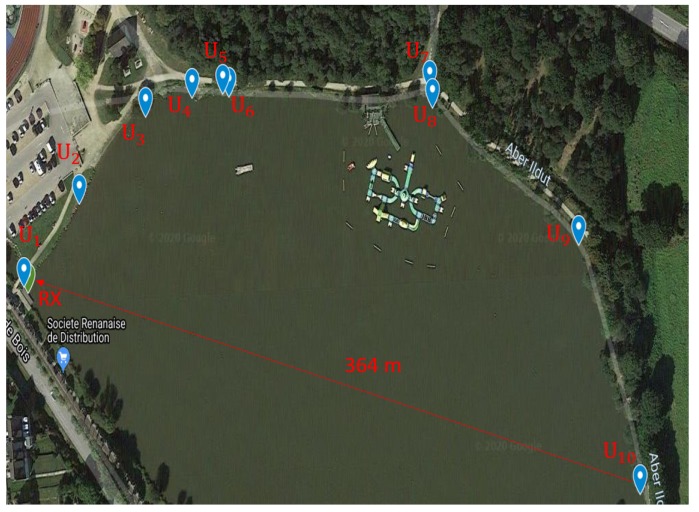
Experiment scheme on the Ty-Colo lake of St-Renan.

**Figure 10:**
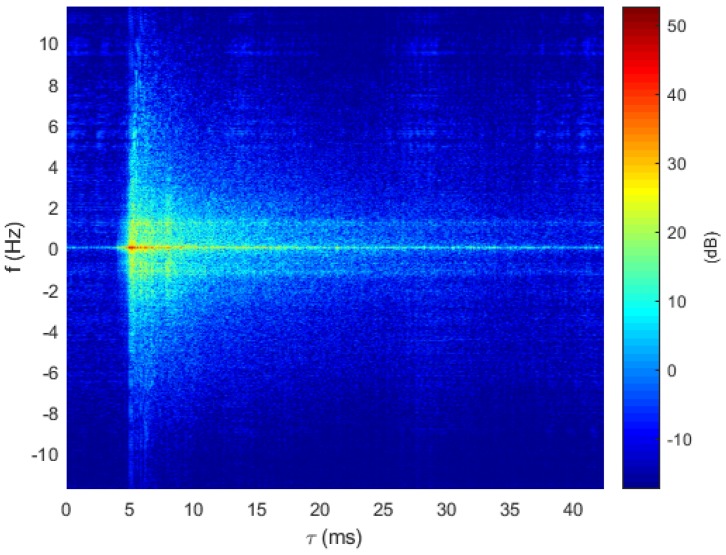
Delay–Doppler spread function for the Ty-Colo lake.

The sounding experiments took place in July 2019 at the lake of Ty-Colo, Saint Renan, France. The depth of the lake is around 5 m, and up to 10 transmission ranges between [47,364] m were sounded successively with one hydrophone at the receiver side, as depicted in Figure 9. Each channel sounding was performed over 3 min and 30 s, using a 255 Maximal Length Sequence (MLS) probe signal [27] centered on $f_{c}=27$ kHz over a 6 kHz bandwidth. Figure 10 provides an example of the delay–Doppler spread extracted from the successive estimated Channel Impulse Response (CIR). Estimated channel delay spreads and Doppler spreads are reported in Table 3.

### 5.2. Watermark Replay Channel

To simulate a real experiment, we consider in this section the Watermark channel [17], which is a replay channel simulator driven by measurements of the time-varying CIR. The principle of the simulator consists of distorting input waveforms by convolving them with measured channels. To simulate a multiuser communication, we sum the output of several Watermark channels fed by different CIRs and delayed by the relative range of each user. The operation of the channel replay for a static multiuser communication in the Single Input Single Output (SISO) case can be expressed in baseband as:

(25) $$r\left(t\right)=\sum_{i=1}^{N_{u}}\int_{-\infty }^{+\infty }{\hat{h}}_{i}\left(\tau ,t\right)s_{i}\left(t-\tau -{\bar{\tau }}_{i}\right)d\tau +n\left(t\right),$$

where $s_{i}\left(t\right)$ is the input signal, ${\hat{h}}_{i}\left(\tau ,t\right)$ is the recorded CIR of the *i*-th user, ${\bar{\tau }}_{i}$ is communication delay between the *i*-th user and the receiver, and n(t) is Gaussian noise.

For mobile multiuser communication, the Doppler shift is simulated by resampling and phase-rotating the transmitted signal as follows:

(26) $$r\left(t\right)=\sum_{i=1}^{N_{u}}\int_{-\infty }^{+\infty }{\hat{h}}_{i}\left(\tau ,t\right)s_{i}\left(\left(1-a_{i}\right)\left(t-\tau -{\bar{\tau }}_{i}\right)\right)e^{j2\pi f_{c}a_{i}\left(t-\tau \right)}d\tau +n\left(t\right).$$

In the following, the Doppler shift will be known by the receiver and compensated by the relation (A1). The Ty-Colo lake channel parameters are summarized in Table 3, whereas the transmission system parameters are provided in Table 2.

### 5.3. Performance Results

#### 5.3.1. Static Users

**Figure 11:**
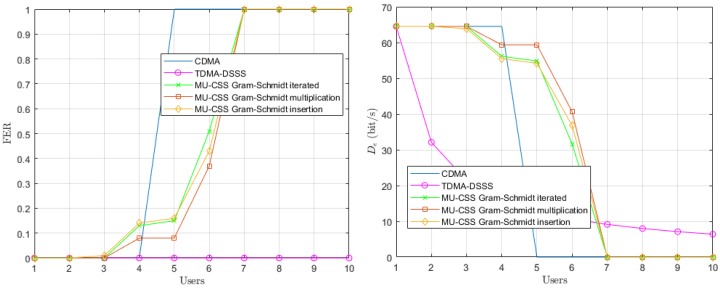
Average FER performance (**left**) and effective data rate per user (**right**) versus number of users for the Ty-Colo lake with the replayed channel with static users.

Figure 11 provides the performance of multiuser transmission techniques over the Watermark channel fed by the Ty-Colo lake channel soundings. It can be noticed that the FER, and consequently the effective data rates, are worse than in the simulation. This can mainly be explained by the fact that the experimental soundings are in very shallow water (≈5 m), leading to a much more important multipath effect and, as a consequence, to higher multiple-access interference terms. Meanwhile, the FER performance of MU-CSS transmissions are still better than CDMA ones with up to six simultaneous users (except the case $N_{u}=4$, where the CDMA is slightly ahead). Beyond this threshold, TDMA transmission is more suitable, despite its low data rate due to a large number of users.

#### 5.3.2. Mobile Users

**Figure 12:**
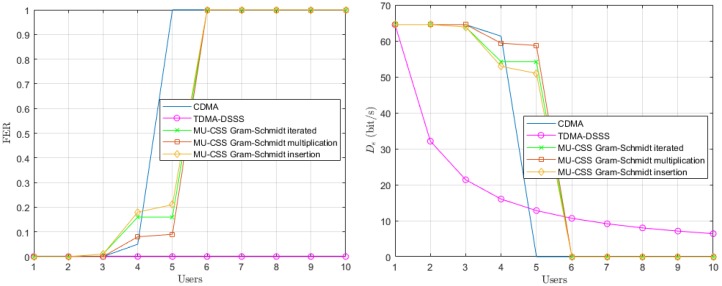
Average FER performance versus number of users for the Ty-Colo lake with the replayed channel with mobile users (**left**), and average effective data rate per user versus number of users for the Ty-Colo lake with the replayed channel with mobile users (**right**).

In Figure 12, the AUVs’ motion is emulated by adding a motion-induced Doppler scale at the output of the Watermark channel. For each frame, the speed value of each AUV is randomly selected in the interval of [−2,2] m/s. We can see that the performances of all access schemes are degraded, except for TDMA. With one to six users, the MU-CSS transmissions remain globally more interesting in terms of the effective data rate. As seen in the simulation, the MU-CSS with the Gram–Schmidt insertion method is confirmed in experiments to provide the highest robustness of all of the MU-CSS construction methods. Beyond six users, the TDMA is demonstrated to be more advantageous.

## 6. Conclusions and Future Works

In this paper, we have proposed a new multiuser transmission technique based on a HFM signal, denoted MU-CSS, in the context of UWA communication within an AUV fleet. By using the Gram–Schmidt orthogonalization, we derived three construction methods for MU-CSS, allowing a very simple matched filter decoding scheme at the receiver side. Simulation comparisons against traditional CDMA, with a single user decoding over static and time-varying shallow water UWA models, demonstrate a superior effective data rate for the proposed MU-CSS scheme, even if the number of users is large and even if the users are in motion, as for an AUV fleet. The experimental results with the Watermark channel replay fed by channel soundings confirm the superiority of MU-CSS transmissions in a realistic scenario. The MU-CSS is demonstrated to be globally superior to CDMA for up to six users. Furthermore, the traditional TDMA approach is demonstrated to be more efficient. The MU-CSS approach, especially when associated with the Gram–Schmidt construction method, offers a set of waveforms that provide good orthogonal properties even in the UWA uplink channel, so that such waveforms do not require a complex multiuser decoding scheme at the receiver side. Thereby, MU-CSS transmission techniques constitute an interesting alternative to asynchronous CDMA for UWA networks.

In a future work, we will consider multi-channel decoding for MU-CSS in order to improve the number of users to be simultaneously correctly decoded, and also take into account real Doppler-shift estimation and its impact on decoding performance when AUVs have different speeds and directions.

## Figures and Tables

**Table 1:** Underwater Acoustic (UWA) channel model parameters.

Symbol	Signification	Value
$f_{c}$	Center frequency	23 kHz
$N_{u}$	Number of AUVs	[1,10]
$f_{s}$	Sample frequency	100 kHz
*B*	Signal bandwidth	4 kHz
$D_{i}$	Transmission range	[0.1,1] km
$z_{w}$	Water depth	10 m
$\tau_{max}$	RMS channel delay spread [20]	[0.52,0.84] ms
SNR	Signal to noise ratio	10 dB
$v_{i}$	User relative speed	[−2,2] m/s
$\sigma_{a}$	Residual motion-induced Doppler spread standard deviation	$10^{-5}$

**Table 2:** System parameters.

Symbol	Signification	Value
*M*	Modulation order	2 (DBPSK)
$N_{s}$	Number of symbols per frame	200
$N_{f}$	Number of frames	5000
C	FEC code type	Convolutive code
$g_{C}$	FEC code generator	(${133,171)}_{o}$
$R_{C}$	FEC code rate	$\frac{1}{2}$
$T_{g}$	Guard interval time	15 ms
$T_{h}$	Duration of the chirp signal	7.75 ms
$T_{c}$	Chip duration	0.25 ms
$N_{SF}$	PN length code	31
$N_{IT}$	Number of iterations	1000
*p*	Insertion step	7
α	Pulse shaping filter roll-off factor	0.25
$T_{s}$	Symbol duration	7.75 ms

**Table 3:** Watermark channel parameters.

Symbol	Signification	Value
$f_{c}$	Center frequency	27 kHz
$f_{s}$	Sampling frequency	96 kHz
*B*	Signal bandwidth	4 kHz
$D_{i}$	Transmission range	[47,364] m
$z_{w}$	Water depth	5 m
SNR	Signal-to-noise ratio	10 dB
$\tau_{max}$	RMS channel delay spread [20]	[4.31,7.27] ms
$\sigma_{max}$	RMS channel Doppler spread [20]	[0.86,2.51] Hz

## Appendix A. Calculation of *γ_i,k_*, *η_i,k_* and *w_i,k_*

The received baseband signal after Doppler pre-processing can be expressed as:

(A1) $$\begin{array}{c} z_{i}\left(t\right)=r\left(\frac{t}{1-a_{i}}\right)e^{-j2\pi f_{c}\left(\frac{a_{i}}{1-a_{i}}\right)t}\\ =\left(\sum_{j=1}^{N_{u}}\int_{-\infty }^{+\infty }h_{j}\left(\tau ,\frac{t}{1-a_{i}}\right)s_{j}\left(\left(1-a_{j}\right)\left(\frac{t}{1-a_{i}}-\tau \right)\right)e^{j2\pi f_{c}a_{j}\left(\frac{t}{1-a_{i}}-\tau \right)}d\tau \right)e^{-j2\pi f_{c}\left(\frac{a_{i}}{1-a_{i}}\right)t}\\ +n\left(\frac{t}{1-a_{i}}\right)e^{-j2\pi f_{c}\left(\frac{a_{i}}{1-a_{i}}\right)t}. \end{array}$$

Combination of (2) and (7) yields:

(A2) $$\gamma_{i,k}=\int_{\frac{-T_{s}}{2}}^{\frac{T_{s}}{2}}\int_{-\infty }^{+\infty }h_{i}\left(\tau ,\frac{t+kT_{s}}{1-a_{i}}\right)g_{i}^{*}\left(t\right)g_{i}\left(t-(1-a_{i})\tau \right)e^{-j2\pi f_{c}a_{i}\tau }d\tau dt$$

(A3) $$\begin{array}{c} \eta_{i,k}=\sum_{\begin{array}{c} n=1\\ n\neq k \end{array}}^{N_{s}}d_{i,n}\int_{\frac{-T_{s}}{2}}^{\frac{T_{s}}{2}}\int_{-\infty }^{+\infty }h_{i}\left(\tau ,\frac{t+kT_{s}}{1-a_{i}}\right)g_{i}^{*}\left(t\right)g_{i}\left(t-\tau -\left(n-k\right)T_{s}\right)e^{-j2\pi f_{c}a_{i}\tau }d\tau dt\\ +\sum_{\begin{array}{c} j=1\\ j\neq i \end{array}}^{N_{u}}\sum_{n=1}^{N_{s}}d_{j,n}\int_{\frac{-T_{s}}{2}}^{\frac{T_{s}}{2}}\int_{-\infty }^{+\infty }h_{j}\left(\tau ,\frac{t+kT_{s}}{1-a_{i}}\right)g_{i}^{*}\left(t\right)g_{j}\left(\left(1-a_{j}\right)\left(\frac{t+kT_{s}}{1-a_{i}}-\tau \right)-nT_{s}\right)e^{-j2\pi f_{c}\left(\frac{a_{i}-a_{j}}{1-a_{i}}\left(kT_{s}+t\right)+a_{j}\tau \right)}d\tau dt \end{array}$$

and

(A4) $$w_{i,k}=e^{-j2\pi f_{c}\frac{a_{i}}{1-a_{i}}kT_{s}}\left(\int_{\frac{-T_{s}}{2}}^{\frac{T_{s}}{2}}g_{i}^{*}\left(t\right)n\left(\frac{t+kT_{s}}{1-a_{i}}\right)e^{-j2\pi f_{c}\left(\frac{a_{i}}{1-a_{i}}\right)t}dt\right)$$

## Appendix B. Justification of the MU-CSS Gram–Schmidt Construction Process

To have the orthogonality between the different $e_{i}\left(t\right)$, we use a variant of the Gram–Schmidt process [25], which is a method for orthogonalizing a set of vectors in an inner product space. The inner product is defined by $\forall f,g\in L^{2}\left(R\right)$ as $\langle f\left(t\right),g\left(t\right)\rangle =\int_{\frac{-T_{s}}{2}}^{\frac{T_{s}}{2}}f\left(t\right)g^{*}\left(t\right)dt$. Let $\left\{c_{1}\left(t\right),c_{2}\left(t\right)\right\}$ be a set of linearly independent vectors. We add the vector $e_{0}\left(t\right)$ to the previous family and we build an orthogonal family from vector $e_{0}\left(t\right)$. By the Gram–Schmidt process, we have:

(A5) $$e_{1}\left(t\right)=c_{1}\left(t\right)+\alpha_{1}e_{0}\left(t\right).$$

Using orthogonality, the previous equation gives:

(A6) $$\begin{array}{cc} \; & \langle c_{1}\left(t\right),e_{0}\left(t\right)\rangle +\alpha_{1}\left|\right|e_{0}\left(t\right){\left|\right|}_{2}^{2}=0 \end{array}$$

(A7) $$\begin{array}{cc} \Leftrightarrow & \alpha_{1}=-\frac{\langle c_{1}\left(t\right),e_{0}\left(t\right)\rangle }{\left|\right|e_{0}\left(t\right){\left|\right|}_{2}^{2}}=-\frac{\int_{\frac{-T_{s}}{2}}^{\frac{T_{s}}{2}}c_{1}\left(t\right)e_{0}^{*}\left(t\right)dt}{\left|\right|e_{0}\left(t\right){\left|\right|}_{2}^{2}}. \end{array}$$

For the last vector, the Gram–Schmidt process gives:

(A8) $$e_{2}\left(t\right)=c_{2}\left(t\right)+\beta e_{0}\left(t\right)+\alpha_{2}e_{1}\left(t\right).$$

We take β=0 because that is enough to have orthogonality, and we obtain:

(A9) $$e_{2}\left(t\right)=c_{2}\left(t\right)+\alpha_{2}e_{1}\left(t\right).$$

Using orthogonality, the previous equation becomes:

(A10) $$\begin{array}{cc} \; & \langle c_{2}\left(t\right),e_{1}\left(t\right)\rangle +\alpha_{2}\left|\right|e_{1}\left(t\right){\left|\right|}_{2}^{2}=0 \end{array}$$

(A11) $$\begin{array}{cc} \Leftrightarrow & \alpha_{2}=-\frac{\langle c_{2}\left(t\right),e_{1}\left(t\right)\rangle }{\left|\right|e_{1}\left(t\right){\left|\right|}_{2}^{2}}=-\frac{\int_{\frac{-T_{s}}{2}}^{\frac{T_{s}}{2}}c_{2}\left(t\right)e_{1}^{*}\left(t\right)dt}{\left|\right|e_{1}\left(t\right){\left|\right|}_{2}^{2}} \end{array}$$

By generalization, we deduce the Equation (10).
